# DNA Damage and Repair in Degenerative Diseases 2016

**DOI:** 10.3390/ijms18010166

**Published:** 2017-01-16

**Authors:** Guillermo T. Sáez

**Affiliations:** Department of Biochemistry and Molecular Biology, Faculty of Medicine and Odontology, Instituto de Investigación Sanitaria, Hospital Clínico de Valencia (INCLIVA), Service of Clinical Analysis, University Hospital Dr. Peset. University of Valencia, Avda, Blasco Ibañez 15, 46010 Valencia, Spain; Guillermo.saez@uv.es; Tel.: +34-96-3864160

Given the great importance of the integrity of DNA for the correct transmission of the genetic message, repairing the induced lesions to its molecular structure by different endogenous or exogenous origin is crucial for the maintenance of homeostasis and biological functions of living organisms. The complete genome of a cell takes between hours and a few days to reproduce, and dysregulation of DNA repair plays a key role in genomic instability. The repair of genetic material is particularly relevant if we consider that the frequency of injuries is about 10^4^–10^5^ impacts per cell and per day. Therefore, cells must have evolved to deal with DNA damage. These lesions, if not repaired, can alter the expression of proteins, including transcriptional factors and, as a consequence, the normal regulation of molecular signaling pathways, resulting in diverse pathological processes of considerable severity. Accumulation of DNA damage, affecting each of the approximately 10^13^ cells in the human body, can lead to multiple diseases, such as neurodegenerative disorders, cancers, immune deficiencies, infertility and even aging. In recent years, the information obtained about the implications of genetic material alteration and its repair, as a cause of a wide spectrum of degenerative diseases, has grown exponentially and represents an aspect of great interest for biomedical and translational research. In this sense, it has been very useful to develop new methods of study; the description of the inductors’ mechanisms of injury; the identification of specific biomarkers; the response and efficiency of repair systems; and the clinical situations related with an inefficient repair of damaged DNA. The relevance of these advances for biomedical sciences is highlighted by the 2015 Nobel Prizes for Chemistry awarded to Drs. Tomas Lindahl, Paul Modrich and Aziz Sancar for their life-long work characterizing the three DNA repair pathways, known as base excision repair (BER), mismatch repair (MMR) and nucleotide excision repair (NER). These studies have also been complemented by prestigious investigators who resolved the mechanisms involved in the repair of double-stranded breaks (DSBR) by homologous recombination (HR) and non-homologous end joining (NHEJ), DNA–DNA and DNA–protein crosslinks (DPCs) and the incorporation of ribonucleotide into DNA. BER is achieved by lesion-specific DNA glycosylases. These enzymes cleave the N-glycosidic bond and remove the damaged bases, leaving as a result an abasic or AB site that is later repaired by the apurinic/apyrimidinic endonuclease 1 (AP1) enzyme. A specific repair enzyme serves to remove ROS-induced oxidized guanines (8-oxo-dG) from DNA, known as the 8-oxo-guanine glycosilase 1 or hOGG1 in humans. Inappropriate incorporation of uracil into DNA is recognized by a uridine DNA glycosylase (UDG), first identified in E.coli in 1974, when the discovery of this repair pathway took place.

NER is a versatile and more complex repair mechanism, specialized in the removal of bulky, helix-distorting lesions from DNA, such as cyclobutane pyrimidine dimers (CPD) andphotoproducts, commonly produced by ultraviolet UV radiation. A multi-enzyme system requiring some thirty different proteins participates in the excision of short single-strand polynucleotide segments from damaged DNA. The MMR system is essential for the repair of misincorporated DNA, bases during DNA replication, that have escaped the proofreading activity of polymerases. In humans, the MMR pathway is carried out by the major protein complexes known as MutS and MutL, based on their homology to the MMR proteins in E.coli. The MMR pathway comprises recognition and excision steps where the error-containing strand is degraded, leaving a gap, which is further filled by DNA synthesis.

In mammalian cells, the two main mechanisms responsible of DSBs repair are homologous recombination (HR) and non-homologous end-joining (NHEJ). HR repair is an error-free mechanism restricted to the late-S and G2 phases of the cell cycle. NHEJ is an error-prone mechanism that operates all along the cell replication cycle. Both DNA interstrand crosslinks and DPCs seem to share some of their repair mechanisms or at least use common elements. The (NER) enzyme heterodimer formed by the excision repair cross complementation group 1 (ERCC1) and the xeroderma pigmentosum group F (XPF), have been shown to be involved in the repair of some types of DNA interstrand crosslinks as well as DPCs. For a review, see [1].

These specific mechanisms of DNA repair are activated according to the nature of DNA damage, which implicates a plethora of metabolic, signal transduction pathways and regulatory mechanisms, where reactive oxygen species (ROS), together with other environmental situations, seem to act as important initiation factors. Genetic and epigenetic factors influencing the normal regulation of cell cycle, cell death and the response to therapy of tumor cells, receive great attention and are a matter of increasing interest in cancer research.

In this 2016 issue, we have collected the last original contributions and reviews of different researchers in this experimental field that reflect the progress made and the future of this scientific area in the biomedical world. As expected, most of the reviews and original scientific contributions deal with the mechanisms of DNA damage and the modulation of its repair systems as protagonists of the tumorogenic scenario.

DNA damage response (DDR) is a complex mechanism involving a long and growing list of different molecules and the roles of the new “dots”; the potential mechanisms in the connection between DDR and DNA repair and in the promotion of physiological or pathological aging was also reviewed [2].

In the paper by IkeuchI et al., the authors suggest that the oncogene v-Src induces chromosome bridges in a caffeine-sensitive manner by generating DNA damage and that this effect may be related with the malignant progression of cancer cells [3].

On the other hand, the modulation of DDR by the Kaposi sarcoma-associated herpes virus (KSHV), through the activation of the ataxia telangiectasia mutated (ATM) pathway and the phosphorylation of the tumor suppressor protein p53, has been proposed to be involved in the malignant transformation of infected cells [4].

New ideas, in relation with the mechanisms underlying the response to DNA damage, in terms of cell cycle checkpoints and apoptosis after the exposure to cancer therapeutic agents, were also reported by Mirzayans et al., emphasizing the role of caspase 3 and the stimulation of prostaglandin E secretion in tumor cells repopulation [5].

Epigenetic changes, including the hypomethylation of gene promoters, lead to the ectopic expression of a large number of proteins, normally restricted to the germ cells of the testis. The oncogenic and replication stress, through the genomic instability induced by these germ cell proteins in the development of cancer, was reviewed by Yoheswaran et al. and presented as promising targets for novel therapeutic strategies [6].

Recent studies have proven that genetic and epigenetic factors can alter the DNA damage response and repair that may have profound effects on the efficiency of radiation and chemotherapy treatment of different tumors. The role of genetic polymorphisms, as well as the regulation of epigenetic factors such as miRNAs and lncRNAs on DNA damage repair in response to radio and chemotherapy in non-small cell lung cancer (NSCLC), was addressed and reviewed by lI et al. showing a new view and suggesting future possibilities for individual tumor treatment [7].

Current information supports the role of miRNAs expression as effective regulators of DNA damage repair. The paper by Encarnación et al. shows a significant difference in the expression of plasma miRNA Let-7b in breast cancer patients with high DNA repair capacity, suggesting a possible role of Let-7b in DNA repair through the NER during breast carcinogenesis. This study also sheds light on the conflicting roles of let-7b expression in terms of breast cancer risk reported in several published studies [8].

As reviewed by Mihoko Kai, the recently discovered gene by her group, RBM14, which contributes to glioblastoma multiform (GBM)’s treatment resistance, is known to function in the transcription and RNA splicing and to regulate the DNA-PK-dependent non-homologous end-joining pathway through its recruitment to DNA double-strand breaks (DDBs) in a PARP1-denpendent manner [9]. According to the report by Verver et.al, NSMCE2 appeared not essential for a proper DNA damage response or cell survival after DSB induction by ionizing irradiation (IR). Interestingly, by way of immunoprecipitations (IPs) and mass spectrometry studies, they found that the SMC5/6 complex physically interacts with the DNA topoisomerase IIα(TOP2A) and propose that the SMC5/6 complex functions in resolving TOP2A-mediated DSB-repair intermediates generated during replication [10].

The development of new radiobiological and diet intervention strategies and their application for the treatment of tumor diseases is a clear example of translational research of great priority, which represents an attractive area in cancer research. The mechanisms of killing human epidermal growth factor receptor 2 (HER2) positive cells by using radioimmunotherapy(RIT) in combination with 177Lu-trastuzumab are presented as an elegant design in oncology therapy. 177Lu-trastuzumab induces cell death via DNA double strand breaks (DSB), caspase-3 apoptosis, and reduction of DNA-PK expression, which is associated with the repair of DNA non-homologous end joining damage. According to the results of Young et al., 177-trastuzumab is an effective therapeutic strategy for the management of intraperitoneal tumor diseases [11].

In the review by Turinetto et al. and collaborators, the importance of the senescence-associated distrurbances of human mesenchymal stem cells (hMSCs), and the evaluation of the possible mechanisms for its prevention, was emphasized as a useful strategy to achieve efficient cell-based therapeutic approaches [12].

The ω-3 docosahexaenoic acid (DHA) is a polyunsaturated fatty acid (PUFA) that shows anticancer activity by inducing apoptosis of some human cancer cells. DHA induces oxidative stress and oxidative DNA adducts formation by depleting intracellular glutathione (GSH) and decreasing the mitochondrial function of cancer cells. It has been shown that DHA affects DNA repair processes, including DNA-dependent protein kinases and mismatch repair in cancer cells. Moreover, as reviewed by Song et al., DHA enhanced the efficacy of anticancer drugs by increasing drug uptake and suppressing survival pathways in cancer cells [13].

In addition to PUFA, other dietary bioactive compounds such as different polyphenols-related metabolites, seem to reveal profound implications on the normal function and regulation of the DNA damage and DNA damage response (DNA/DDR) network. The in silico analysis used by the Teodori’s group allowed the identification of pathways shared by different miRs and the demonstration of how miRs -146, and -21 play a central role in the interplay among DD/DDR and the bioactive compounds. This observation may provide the means to assess the antiaging and chemopreventive properties of specific dietary compounds [14].

In addition to cancer, the inefficient repair of DNA has been shown to play an important role in the pathogenicity of other degenerative diseases. In the review by Ranchoux et al., high levels of DNA damage were reported to occur in both human and animal models of pulmonary arterial hypertension (PAH). Impaired DNA-response mechanisms may lead to an increased mutagen sensitivity in PAH patients and has been related with a decreased expression of breast cancer 1 protein (BRCA1) and DNA topoisomerase 2-binding protein 1 (TopBP1) which are involved in maintaining genome integrity [15].

Oxidative stress-induced DNA damage and deficient repair of oxidative DNA lesions have been proposed to contribute to the development of schizophrenia and autism spectrum disorder (ASD). The review by Markkanen et al. summarizes the current evidence of cancer comorbidity in these brain disorders and discusses the putative roles of oxidative stress, DNA damage and DNA repair in the aetiopathology of schizophrenia and ASD [16].

Parkinson’s disease is another and well established clinical situation in which oxidative stress plays an important role and as a result DNA became damaged. The expression of phosphatase and tensin homolog on chromosome 10 (PTEN) appears to be a determinant of the neuronal cell death and therefore a potential molecular target of novel pharmacologic interventions. Ogino and cols. reviewed these aspects as well as the implications of hormone signaling pathways in the regulation of DNA damage response and repair, providing a broad interpretation on the molecular mechanisms for treatment of this neurodegenerative disease [17].
